# Motor Learning in Healthy Humans Is Associated to Gray Matter Changes: A Tensor-Based Morphometry Study

**DOI:** 10.1371/journal.pone.0010198

**Published:** 2010-04-15

**Authors:** Massimo Filippi, Antonia Ceccarelli, Elisabetta Pagani, Roberto Gatti, Alice Rossi, Laura Stefanelli, Andrea Falini, Giancarlo Comi, Maria Assunta Rocca

**Affiliations:** 1 Neuroimaging Research Unit, Institute of Experimental Neurology, Division of Neuroscience, Scientific Institute and University Hospital San Raffaele, Milan, Italy; 2 Department of Neurology, Scientific Institute and University Hospital San Raffaele, Milan, Italy; 3 Laboratory of Movement Analysis, School of Physiotherapy, Scientific Institute and University Hospital San Raffaele, Milan, Italy; 4 Department of Neuroradiology, Scientific Institute and University Hospital San Raffaele, Milan, Italy; Cuban Neuroscience Center, Cuba

## Abstract

We used tensor-based morphometry (TBM) to: 1) map gray matter (GM) volume changes associated with motor learning in young healthy individuals; 2) evaluate if GM changes persist three months after cessation of motor training; and 3) assess whether the use of different schemes of motor training during the learning phase could lead to volume modifications of specific GM structures. From 31 healthy subjects, motor functional assessment and brain 3D T1-weighted sequence were obtained: before motor training (time 0), at the end of training (two weeks) (time 2), and three months later (time 3). Fifteen subjects (group A) were trained with goal-directed motor sequences, and 16 (group B) with non purposeful motor actions of the right hand. At time 1 *vs*. time 0, the whole sample of subjects had GM volume increase in regions of the temporo-occipital lobes, inferior parietal lobule (IPL) and middle frontal gyrus, while at time 2 *vs*. time 1, an increased GM volume in the middle temporal gyrus was seen. At time 1 *vs*. time 0, compared to group B, group A had a GM volume increase of the hippocampi, while the opposite comparison showed greater GM volume increase in the IPL and insula in group B *vs*. group A. Motor learning results in structural GM changes of different brain areas which are part of specific neuronal networks and tend to persist after training is stopped. The scheme applied during the learning phase influences the pattern of such structural changes.

## Introduction

Learning motor skills is associated with an increased spatial and temporal accuracy of movements with practice and a reduction of attention to execute actions [1], [2]. Several functional neuroimaging studies have identified a set of brain regions showing dynamic changes in their profiles of activations during different stages of motor learning [1], [2]. More recently, longitudinal voxel-based morphometry (VBM) studies have shown that structural changes of the gray matter (GM) do also occur following motor learning in healthy adult subjects [3], independently of their age [4]. Such structural modifications are supposed to occur relatively early during the learning process of new motor skills, since they have been observed even after seven days of daily training [5]. Although the neurobiological substrates underlying these brain structural changes are largely unknown, exercise-induced increases in hippocampal cerebral blood flow, measured with MRI, were found to correlate with postmortem measurements of neurogenesis [6]. In addition, sprouting of new connections, dendritic spine growth, and modification in the strength of existing connections are all likely to explain at least part of the observed structural MRI changes [7], [8].

Tensor-based morphometry (TBM) infers volume modifications from the non-linear deformation field required to warp two serial MRI scans, thus allowing voxel-wise longitudinal volumetric differences to be detected [9]. Such a technique, has been applied to track patterns of atrophy progression in various neurodegenerative disorders [10], [11], [12], [13], [14], [15], [16], and, more recently, in young healthy individuals during cognitive learning [17]. Against this background, we used TBM to map changes of GM volume associated with motor learning following two weeks of daily motor training of the dominant right hand in a group of young healthy individuals. Since preliminary studies suggested that GM volume changes might be transient [5], [18], all the subjects were re-assessed behaviorally and with structural MRI three months after the cessation of motor training.

Studies assessing morphological changes of brain structures during motor learning have been mainly focused on juggling, a complex visuo-motor integration task. Learning fine finger motor skills requires the repetition of fixed sequences of movements [19] and has been associated with changes of activation of several brain areas, mainly located in the fronto-parietal lobes. Behavioral and kinematic studies in healthy [20] and diseased [21] people provided evidence that the presence of objects, during motor learning, might improve motor performance. In line with this, object-related actions have been consistently demonstrated to recruit specific neural networks [22], [23], [24]. As a consequence, we also assessed whether the use of different schemes of motor training (one based on training of transitive, object-related and goal-directed motor sequences, and the other on training of intransitive non purposeful motor actions) was associated to different patterns of GM structural modifications.

## Results

Table 1 shows the Purdue Pegboard Test (PPT) performance at each study time point in the two groups of subjects. No between-group difference was found in the performance of the manual and assembly test subsets at any time point. Both groups showed a significant improvement of PPT performance at time 1 *vs*. time 0 (manual PPT: group A, p = 0.02, group B, p = 0.0001; assembly PPT: group A, p = 0.0001, group B, p = 0.003), while no performance difference was found at time 2 *vs*. time 1.

**Table 1:** Purdue Pegboard Test performance (mean ± standard deviation) in the two groups of subjects at each study time point.

Purdue Pegboard Test *Mean (SD)		Group A	Group B
**Manual**	**Time 0**	17.6 (1.3)	17.4 (1.7)
	**Time 1**	18.4 (1.6)	18.6 (1.6)
	**Time 2**	18.9 (1.3)	18.9 (1.7)
**Assembly**	**Time 0**	32.2 (2.6)	32.5 (3.0)
	**Time 1**	35.0 (3.0)	34.8 (3.8)
	**Time 2**	35.6 (2.7)	35.7 (3.9)

*Number of pegs placed in 30 sec.

Group A = training with repetitive, transitive, object-related and goal-directed motor sequences of the right hand; group B = training with intransitive non purposeful motor actions of the right hand.

Time 0 = baseline evaluation (on the day of the beginning of the motor training), time 1 =  at the end of the motor training (two weeks), time 2 = 3 months after cessation of motor training.

See text for further details.

All subjects had normal brain MRI dual-echo scans. At baseline, no GM differences were found between subjects of group A and those of group B (p<0.05, family-wise error [FWE] corrected).

The analysis performed with a smoothing of 8-mm and 12-mm gave similar results, with larger clusters at 12-mm. As expected, a few additional, small clusters were seen with a 8-mm Gaussian kernel. However these clusters did not survive correction for multiple comparisons. As a consequence, we chose to report the results obtained with a 12-mm Gaussian kernel.

### a) Within-group changes of GM volumes

**Figure 1:**
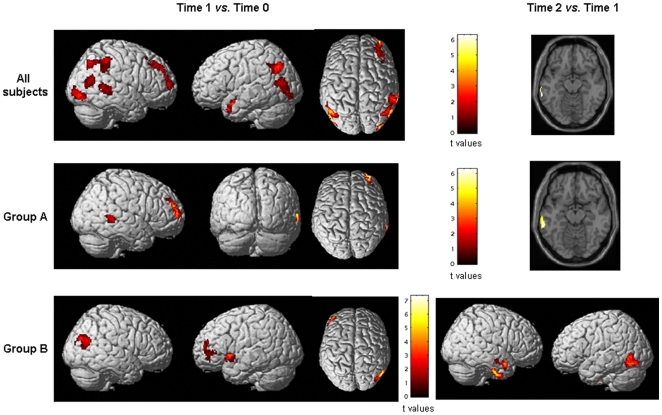
Statistical parametric mapping (SPM) regions (color-coded for t values) (one-sample t test, p<0.05, corrected for multiple comparisons, family-wise error), superimposed on high-resolution T1-weighted scans, where increases of gray matter (GM) volume at time 1 *vs.*time 0 and time 2 *vs*. time 1 were seen in the whole group of subjects (top row), in those who were trained with transitive, object-related and goal-directed motor actions of their right hand (middle row); and in those who were trained with intransitive, non purposeful motor actions of the right hand (bottom row). See text for further details. Images are in neurological convention.

At time 1, compared to time 0, the whole sample of subjects showed significant increases of GM volume (p<0.05 FEW corrected) of the bilateral superior temporal gyrus (STG) (Montreal Neurologic Institute [MNI] space coordinates: left STG −42, 6, −18; t value = 6.95; right STG 68, −44, 4; t value = 5.89), bilateral inferior occipital gyrus (IOG) (BA19) (MNI space coordinates: left IOG −52, −78, 0; t value = 5.80; right IOG 36, −94, −2; t value = 5.71), bilateral middle temporal gyrus (MTG) (BA39) (MNI space coordinates: left MTG −52, −72, 14; t value = 5.02; right MTG 58, −66, 18; t value = 5.54), bilateral inferior parietal lobule (IPL) (BA40) (MNI space coordinates: left IPL −48, −68, 36; t value = 5.41; right IPL 58, −42, 48; t value = 5.26), right middle frontal gyrus (MFG) (BA10) (MNI space coordinates: 34, 62, 8; t value = 5.71) (Figure 1). Using a threshold of p<0.001 uncorrected, GM volume increase was also detected in the left MFG (BA6) (MNI space coordinates: −22, 10, 64; t value = 4.49).

Considering the variations of GM volumes in the two groups of subjects separately, group A showed significant GM volume increases of the right MTG (MNI space coordinates: 68, −44, 2; t value = 5.91), and right MFG (BA10/BA9) (MNI space coordinates: 34, 62, 8 and 42, 42, 34; t values = 6.12 and 4.06) (Figure 1). Group B had significant GM volume increases of the right MTG (BA39) (MNI space coordinates: 58, −66, 18 and 52, −72, 24; t values = 6.16 and 5.36), left insula (MNI space coordinates: −36, 12, −6; t value = 5.57), and left MFG (BA6) (MNI space coordinates: −44, 52, 8; t value = 6.27) (Figure 1). The use of a threshold of p<0.001 uncorrected, allowed to detect GM volume increases of the bilateral STG, bilateral IOG, bilateral IPL and left MTG, in both groups of subjects.

At time 2, compared to time 1, the whole sample of subjects showed a significant increase of GM volume of the left MTG (BA21) (MNI space coordinates: −66,−40,−12; t value = 5.42) (Figure 1). Such a change was detected in both group A and B, when they were assessed separately. In addition, group B showed significant GM volume increases of the left middle occipital gyrus (MOG) (BA19) (MNI space coordinates: −48, −74, −12; t value = 5.58), the right inferior temporal gyrus (ITG) (MNI space coordinates: 56, −14, −38; t value = 5.07), and the right MTG (BA21) (MNI space coordinates: 56, 6, −22; t value = 5.97) (Figure 1). The opposite comparison (GM volume changes at time 1 *vs*. time 2) did not show any significant difference both in the entire sample of subjects and when the two subgroups were analyzed separately.

### b) Between-group comparisons of GM volumes changes

**Figure 2:**
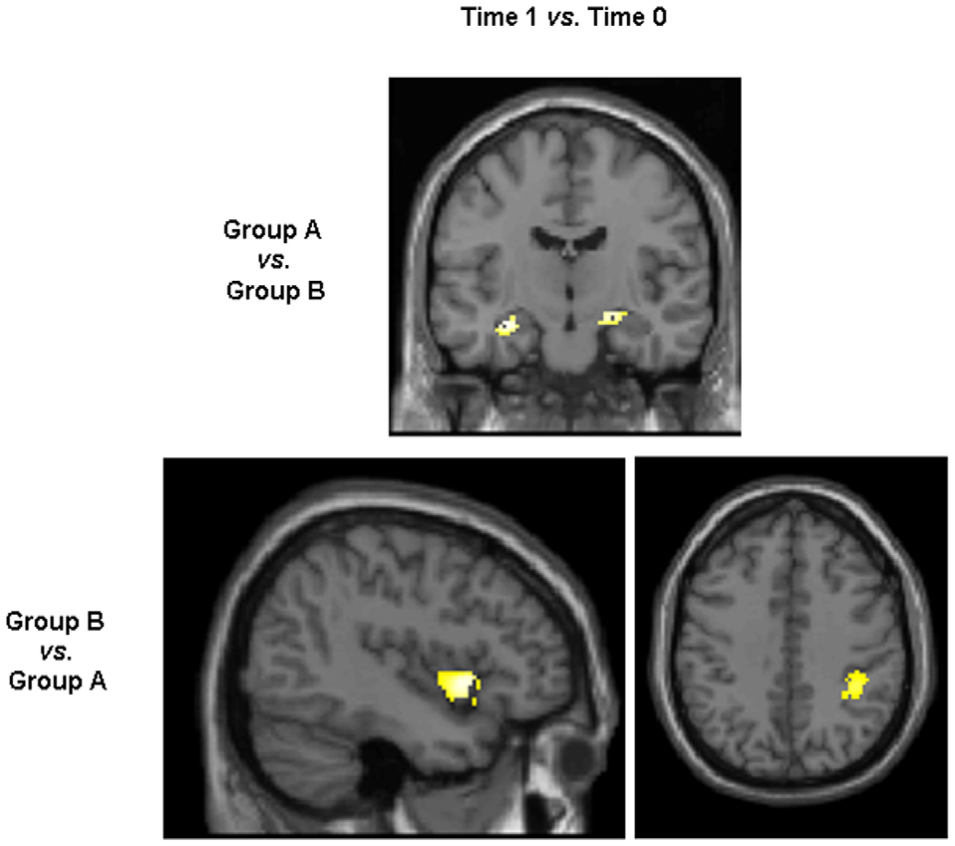
Statistical parametric mapping (SPM) regions, superimposed on high-resolution T1-weighted scans showing areas with significant gray matter (GM) volume changes at the between-group comparison (ANCOVA, p<0.05, corrected for multiple comparisons, family-wise error). Top row: areas of significant GM volume increases in group A *vs*. group B at time 1 *vs*. time 0. Bottom row: areas of significant GM volume increase in group B *vs*. group A at time 1 *vs*. time 0. See text for further details. Images are in neurological convention.

At time 1, compared to group B, group A showed significant GM volume increases of the hippocampi, bilaterally (MNI space coordinates: −30, −14, −16, and 24, −10, −12) (Figure 2). The opposite comparison showed a greater GM volume increase of the left insula (MNI space coordinates: −44, 12, 0), and right IPL (MNI space coordinates: 38, −42, 38) in group B *vs*. group A (Figure 2). At time 2, no differences were found between the two groups.

## Discussion

In this study, we used TBM to assess whether longitudinal changes of GM volumes occur in young, healthy individuals following training of their motor skills. In such a case, to evaluate whether they continue to be detectable after the discontinuation of the motor program, all subjects were reassessed behaviorally and by MRI two weeks and three months later. In addition, we also wished to investigate whether different motor training strategies are associated to different patterns of regional GM volume changes. To this end, we compared the effects of two different schemes of motor training of fine finger movements of the right hand, one based on training of goal-directed motor actions, and the other one based on the execution of non purposeful actions.

In the entire sample of subjects, a two-week daily training of fine motor skills with the dominant right hand resulted in significant GM volume increases of the bilateral STG, IOG, MTG, IPL, and right MFG. Using VBM, previous studies found significant changes of GM volume in healthy individuals following motor training. Draganski et al. [18] showed significant increases of GM volumes of the MTG and IPL in people who had learned to juggle for three months compared to those who did not. These results were confirmed by a second study from the same group [5], which detected an effect of juggling on GM volumes of the temporo-occipital cortex and several other areas of the frontal lobes after 28 days of training. Finally, Boyke et al. reported significant GM volume increases of the MTG, areas located in the frontal lobes (including the anterior cingulum), and left hippocampus after three months of juggling in elderly, healthy individuals [4]. The previous studies [4], [5], [18] hypothesized that the increased GM volume found in the temporo-occipital cortex might be related strictly to the type of the training task (i.e., juggling, which requires an increased interaction between the visuo-motor areas to be performed correctly). Remarkably, we found an effect of motor training on volume changes of this region as well as of other areas located in the parietal and frontal lobes which were independent of the training scheme. Indeed, these changes were detected in both groups of subjects. All the regions that others [4], [5], [18] and ourselves reported to undergo structural changes with training are known to be involved in the control of sensorimotor and cognitive functions. In particular, the dorsal premotor cortex (including the MFG) processes spatial information in the context of movement generation and preparation, via an interaction with the parietal cortex [25].

Contrary to the results of the previous VBM studies [4], [5], [18], which suggested that the observed GM changes were reversible after cessation of training, we found that GM volume increases are still detectable at least three months after training was stopped, since the analysis of GM volume changes at time 1 *vs*. time 2 did not show any significant difference. This is in agreement with the results of a recent study [26], which showed persistent changes of GM and WM architecture in healthy individuals 4 weeks after juggling was terminated. We also found that other areas, mainly located in the temporal and occipital lobes and which are likely to be active in memory consolidation, experienced significant structural GM changes after three months. Several issues need to be considered in an attempt to explain the discrepancies between our and previous results. First, all subjects of our study were exposed to a daily standardized training period, monitored by an expert physiotherapist, while those of the other studies were not followed on a day-by-day basis during their training. Second, motor improvement gained with training persisted at three months in our subjects, while they were lost by the subjects of the previous studies [4], [18]. Third, we used TBM instead of VBM. TBM uses two MR images of the same subject acquired at two different time points. After rigid transformation, it applies a non-linear deformation algorithm to measure morphological differences between two scans. Once these differences are measured on a voxel-wise basis, group analysis can be performed after image deformation to the standard space. Conversely, the VBM approach uses a deformation algorithm to calculate the differences between a single time point MRI obtained from a group of subjects and a reference atlas. Critical to both the approaches is the deformation algorithm applied. Usually, the deformation algorithms used in TBM analysis are more sophisticated to compensate for small morphological differences. Remarkably, even when the same deformation algorithm is used in TMB and VBM studies, the serial application of TBM remains more powerful because longitudinal changes of brain tissue are calculated without the confounding factor of intersubject anatomical differences [9], [27]. Finally, we performed a formal statistical analysis of within- and between-group changes at the different time points, whereas the results of the previous studies [5], [18] were mainly drawn from the behavior of the signal plots of different cerebral areas.

The comparison of GM volume changes between the two groups of subjects, following different training strategies, showed structural modifications of selected brain regions at time 1 *vs*. time 0 only. In particular, compared to subjects of group B, those of group A experienced structural changes of the hippocampi. An increase of hippocampal volume after a goal-oriented training, such as juggling, is in line with the results of Boyke et al. in elderly healthy subjects [4]. Using immunofluorescent labeling on postmortem brains, Eriksson et al. [28] demonstrated that the human hippocampus retains its ability to generate neurons throughout life. In addition, using MRI measurements of cerebral blood volume (CBV), Pereira et al. [6] found that exercise in humans had a primary effect on CBV increase in the dentate gyrus, which is the only subregion that subserves adult neurogenesis.

The opposite contrast showed that, over the course of the study, group B *vs*. group A experienced a selective structural change of the IPL and the insula. The insula has extensive reciprocal connections with many brain sensorimotor areas, as well as with the parietal cortex, temporal cortex, basal ganglia, and thalamus [29]. Parietal areas of the right hemisphere code for spatial attention and contribute to the translation from visuo-spatial to body related information during the acquisition of motor skills [30].

In conclusion, our study suggests that motor learning is associated to structural GM changes in “strategic” brain areas that are part of neuronal networks which are instrumental to different training schemes. Although additional longitudinal studies, possibly in larger groups of subjects, are warranted to elucidate better the temporal dynamics of brain structural changes secondary to motor learning in humans, our results support the notion that such changes are likely to persist over, at least, a relatively short follow up after cessation of motor training. These findings might have important implications for the development of rehabilitation strategies in patients with neurological diseases.

## Materials and Methods

The study was approved by the Ethics Committee of Scientific Institute and University Ospedale San Raffaele, Milan, Italy and a written informed consent was obtained from all subjects prior to study entry, according to the Declaration of Helsinki.

### a) Subjects

We recruited 31 right-handed healthy individuals (M/F = 13/18, mean age = 21.9, range = 19–30 years) with no history of neurological disorders and any other medical conditions, and with no drug or alcohol abuse. Handedness was established using the Edinburgh Handedness Inventory Scale [31]. None of the subjects had particular manual skills (e.g, musicians, athletes, crochet, typewriting, etc.) and they were carefully selected on the basis of their daily activities, in terms of hobbies and amusements. Through a computer-generated sequence, all subjects were randomly assigned to two groups, which underwent two different schemes of motor training during the study period: 15 subjects (group A) (M/F = 6/9, mean age = 21.9, range = 19–29 years) were trained with transitive, object-related and goal-directed motor sequences of their right hand (for instance, juggling, playing a guitar, rolling a drumstick among the fingers, starting from the thumb to the little finger, lifting objects of various dimensions, such as little pearls or strings, with a chopstick, etc.); and 16 subjects (group B) (M/F = 7/9, mean age = 21.9, range = 20–30 years) were trained with intransitive non purposeful motor actions of the right hand (for instance, two finger abducing while one rotating, two fingers flexing while other two extending, etc.). A detailed description of the tasks administered to the two groups of subjects during training is given in Table 2. None of the subjects was able to perform any of the trained exercises before entering the study. All subjects were trained to practice the movements by a physiotherapist for 2 weeks with daily 25-minute practice sessions, excluding the weekend. During the training session, the tasks were kept effortful by increasing their complexity and speed (see Table 2). After these period, subjects were instructed not to practice further their learned skills.

**Table 2:** Description of the tasks administered to the two groups of subjects during motor training.

Group A	Group B
Playing a guitar: increase of the number of strings, increase of the number of fingers used, increase of the speed of execution	Schemes of flexion/extension/rotation of the different fingers: e.g., flexion of forefinger and ring finger; flexion of middle finger and little finger; flexion of forefinger and ring finger plus extension of middle finger and little finger and vice versa; equal to the previous one, plus thumb rotation
Juggling: increase of the number of juggling balls, change of the size of juggling balls	Schemes of abduction of the different fingers: e.g., abduction of forefinger and little finger; abduction of forefinger and middle finger plus ring finger and little finger; equal to the previous one plus thumb rotation
Rolling a drumstick among the fingers: increase of the number of fingers used, increase of the speed of execution	Different schemes of finger tapping of the five fingers, with increasing complexity and increasing speed
Lifting objects of various dimensions: change of the size of the objects, change of the weight of the object, change of the speed of execution	

Group A = training with repetitive, transitive, object-related and goal-directed motor sequences of the right hand; group B = training with intransitive non purposeful motor actions of the right hand.

In all the subjects, behavioral and structural MRI data were acquired at baseline (on the day of the beginning of the motor training) (time 0), at the end of motor training (after two weeks ±1 day) (time 1), and 3 months later (±1 day) (time 2).

Fine motor control and performance of the right hand was evaluated with the different subsets of the PPT [32]. In this test participants are asked to place pegs into the holes of a board. It assesses motor speed and coordination and is sensitive to subtle motor dysfunction [32]. Its outcome measure is the number of pegs placed correctly within 30 seconds with the dominant hand. The PPT consists of two different subsets, manual and assembly test, and is used to asses various types of manual labor by measuring 2 types of dexterity: 1) gross movement of the fingers, hands and arms, and 2) fine finger dexterity necessary in an assembly task.

### b) MRI acquisition

Brain MRI were acquired from all subjects at the three time-points using a 3.0 Tesla scanner (Intera, Philips Medical Systems, Best, The Netherlands). The following sequences were acquired from all subjects: 1) dual-echo turbo spin echo (TSE) (TR = 3500 ms, TE = 24/120 ms; echo train length = 5; flip angle = 150°, 44 contiguous, 3-mm-thick, axial slices with a matrix size = 256×256, and a field of view [FOV] = 240×240 mm^2^), and 2) 3D T1-weighted fast field echo (FFE) (TR = 25 ms, TE = 4.6 ms, flip angle = 30°, 220 contiguous, axial slices with voxel size = 0.89×0.89×1 mm, matrix size = 256×256, and FOV = 230×230 mm^2^). TSE sequences were used to exclude the presence of brain macroscopic abnormalities.

### c) Image analysis and post-processing

VBM and the statistical parametric mapping (SPM5) software (www.fil.ion.ucl.ac.uk/spm) were used to assess differences in GM volume between the two groups of subjects at baseline, following the procedures described elsewhere [27].

TBM, as implemented in SPM5, was used to map changes of regional GM volume over time in the entire sample of subjects and in the two groups of subjects, separately. A comprehensive description of TBM image pre-processing is reported elsewhere [10], [15], [17]. We applied a bias correction to the time 1 T1-weighted scans previously coregistered with the time 0 T1-weighted ones, and to the time 2 T1-weighted scans previously coregistered with the time 1 T1-weighted ones, to make them comparable. A high-dimensional deformation field was then used to warp the corrected early images to match the late ones for each individual subject [27], [33]. The amount of volume change was quantified by taking the determinant of the gradient of deformation at a single-voxel level (Jacobian determinant). The following formula was applied to the segmented GM images obtained from the first scans and the Jacobian determinant maps: (Jacobian value −1) × GM. The resulting product images provide estimates of the GM specific volume changes between the first and the second scans (i.e., time 0 *vs*. time 1, and time 1 *vs*. time 2). Segmented GM images from the late scans (time 1 and time 2 scans) were normalized to GM template in MNI space, and the deformation applied to the product images [34]. Normalized images were smoothed using a 12-mm isotropic Gaussian kernel. The use of such a kernel has been shown to minimize the risk of false positive findings [35]. Normalized, smoothed maps of GM over time for each subject were then entered into the statistical analysis. To exclude from the statistical analysis pixels assigned by the segmentation to GM and white matter (WM) with low probability values and pixels with a low inter-subject anatomical overlay after normalization, GM masks were created by averaging GM normalized maps from all subjects. These masks were thresholded at a value of 0.50 and then used as explicit masks during the statistical analysis. To test whether the use of a 12-mm kernel might have influenced our results, all the post-processing was also performed using a 8-mm isotropic Gaussian kernel.

### d) Statistical analysis

Regional changes of GM volume at baseline and over the follow-up were assessed using the general linear model and the theory of Gaussian fields [36]. Within-group changes of GM volume were assessed using a one sample t test. An analysis of covariance (ANCOVA), corrected for age and sex, was used to compare brain regions showing GM changes over time between the two groups of subjects. A whole brain analysis was performed, with a level of significance of p<0.05, corrected for multiple comparisons (FWE). Coordinates of foci of GM changes within each suprathreshold cluster were produced as MNI coordinates. Anatomical localization of the cerebral areas showing GM changes was defined by an experienced observer, using the Talairach Daemon.

Within- and between-group changes of PPT performance were assessed using a paired t test and an unpaired t test, respectively, and SPSS software.

## References

[1] Doyon J (2008). Motor sequence learning and movement disorders.. Curr Opin Neurol.

[2] Doyon J, Benali H (2005). Reorganization and plasticity in the adult brain during learning of motor skills.. Curr Opin Neurobiol.

[3] Draganski B, May A (2008). Training-induced structural changes in the adult human brain.. Behav Brain Res.

[4] Boyke J, Driemeyer J, Gaser C, Buchel C, May A (2008). Training-induced brain structure changes in the elderly.. J Neurosci.

[5] Driemeyer J, Boyke J, Gaser C, Buchel C, May A (2008). Changes in gray matter induced by learning–revisited.. PLoS One.

[6] Pereira AC, Huddleston DE, Brickman AM, Sosunov AA, Hen R (2007). An in vivo correlate of exercise-induced neurogenesis in the adult dentate gyrus.. Proc Natl Acad Sci U S A.

[7] Sur M, Rubenstein JL (2005). Patterning and plasticity of the cerebral cortex.. Science.

[8] Trachtenberg JT, Chen BE, Knott GW, Feng G, Sanes JR (2002). Long-term in vivo imaging of experience-dependent synaptic plasticity in adult cortex.. Nature.

[9] Leow AD, Klunder AD, Jack CR, Toga AW, Dale AM (2006). Longitudinal stability of MRI for mapping brain change using tensor-based morphometry.. Neuroimage.

[10] Kipps CM, Duggins AJ, Mahant N, Gomes L, Ashburner J (2005). Progression of structural neuropathology in preclinical Huntington's disease: a tensor based morphometry study.. J Neurol Neurosurg Psychiatry.

[11] Chiang MC, Reiss AL, Lee AD, Bellugi U, Galaburda AM (2007). 3D pattern of brain abnormalities in Williams syndrome visualized using tensor-based morphometry.. Neuroimage.

[12] Lee AD, Leow AD, Lu A, Reiss AL, Hall S (2007). 3D pattern of brain abnormalities in Fragile X syndrome visualized using tensor-based morphometry.. Neuroimage.

[13] Lepore N, Brun CA, Chiang MC, Chou YY, Dutton RA (2006). Multivariate statistics of the Jacobian matrices in tensor based morphometry and their application to HIV/AIDS.. Med Image Comput Comput Assist Interv Int Conf Med Image Comput Comput Assist Interv.

[14] Brambati SM, Rankin KP, Narvid J, Seeley WW, Dean D (2009). Atrophy progression in semantic dementia with asymmetric temporal involvement: a tensor-based morphometry study.. Neurobiol Aging.

[15] Brambati SM, Renda NC, Rankin KP, Rosen HJ, Seeley WW (2007). A tensor based morphometry study of longitudinal gray matter contraction in FTD.. Neuroimage.

[16] Agosta F, Gorno-Tempini ML, Pagani E, Sala S, Caputo D (2009). Longitudinal assessment of grey matter contraction in amyotrophic lateral sclerosis: A tensor based morphometry study.. Amyotroph Lateral Scler.

[17] Ceccarelli A, Rocca MA, Pagani E, Falini A, Comi G (2009). Cognitive learning is associated with gray matter changes in healthy human individuals: a tensor-based morphometry study.. Neuroimage.

[18] Draganski B, Gaser C, Busch V, Schuierer G, Bogdahn U (2004). Neuroplasticity: changes in grey matter induced by training.. Nature.

[19] Hikosaka O, Nakamura K, Sakai K, Nakahara H (2002). Central mechanisms of motor skill learning.. Curr Opin Neurobiol.

[20] Wu C, Trombly CA, Lin K, Tickle-Degnen L (2000). A kinematic study of contextual effects on reaching performance in persons with and without stroke: influences of object availability.. Arch Phys Med Rehabil.

[21] Trombly CA, Wu CY (1999). Effect of rehabilitation tasks on organization of movement after stroke.. Am J Occup Ther.

[22] Tunik E, Ortigue S, Adamovich SV, Grafton ST (2008). Differential recruitment of anterior intraparietal sulcus and superior parietal lobule during visually guided grasping revealed by electrical neuroimaging.. J Neurosci.

[23] Lepper M, Massen C, Prinz W (2008). What to do and how to do it: sequence learning of action effects and transformation rules.. Acta Psychol (Amst).

[24] Koski L, Wohlschlager A, Bekkering H, Woods RP, Dubeau MC (2002). Modulation of motor and premotor activity during imitation of target-directed actions.. Cereb Cortex.

[25] Picard N, Strick PL (2001). Imaging the premotor areas.. Curr Opin Neurobiol.

[26] Scholz J, Klein MC, Behrens TE, Johansen-Berg H (2009). Training induces changes in white-matter architecture.. Nat Neurosci.

[27] Ashburner J, Andersson JL, Friston KJ (2000). Image registration using a symmetric prior–in three dimensions.. Hum Brain Mapp.

[28] Eriksson PS, Perfilieva E, Bjork-Eriksson T, Alborn AM, Nordborg C (1998). Neurogenesis in the adult human hippocampus.. Nat Med.

[29] Augustine JR (1996). Circuitry and functional aspects of the insular lobe in primates including humans.. Brain Res Brain Res Rev.

[30] Halsband U, Lange RK (2006). Motor learning in man: a review of functional and clinical studies.. J Physiol Paris.

[31] Oldfield RC (1971). The assessment and analysis of handedness: the Edinburgh inventory.. Neuropsychologia.

[32] Mandell RJ, Nelson DL, Cermak SA (1984). Differential laterality of hand function in right-handed and left-handed boys.. Am J Occup Ther.

[33] Ashburner J, Csernansky JG, Davatzikos C, Fox NC, Frisoni GB (2003). Computer-assisted imaging to assess brain structure in healthy and diseased brains.. Lancet Neurol.

[34] Ashburner J, Friston KJ (1999). Nonlinear spatial normalization using basis functions.. Hum Brain Mapp.

[35] Salmond CH, Ashburner J, Vargha-Khadem F, Connelly A, Gadian DG (2002). Distributional assumptions in voxel-based morphometry.. Neuroimage.

[36] Friston KJ, Holmes AP, Poline JB, Grasby PJ, Williams SC (1995). Analysis of fMRI time-series revisited.. Neuroimage.

